# Soybean- and Lupin-Derived Peptides Inhibit DPP-IV Activity on In Situ Human Intestinal Caco-2 Cells and Ex Vivo Human Serum

**DOI:** 10.3390/nu10081082

**Published:** 2018-08-13

**Authors:** Carmen Lammi, Carlotta Bollati, Simonetta Ferruzza, Giulia Ranaldi, Yula Sambuy, Anna Arnoldi

**Affiliations:** 1 Department of Pharmaceutical Sciences, University of Milan, Via Mangiagalli 25, 20133 Milan, Italy; carlotta.bollati@gmail.com (C.B.); anna.arnoldi@unimi.it (A.A.); 2 CREA-Research Centre for Food and Nutrition, Via Ardeatina 546, 00100 Rome, Italy; simonetta.ferruzza@crea.gov.it (S.F.); giulia.ranaldi@crea.gov.it (G.R.); yula.sambuy@crea.gov.it (Y.S.)

**Keywords:** anti-diabetic activity, bioactive peptides, dipeptidyl peptidase IV, glucose metabolism, sitagliptin

## Abstract

Recent investigations have focused on food-derived peptides as novel natural inhibitors of dipeptidyl peptidase IV (DPP-IV), a new target for diabetes. This study aimed to optimize fast, sensitive, and cost-effective DPP-IV assays in situ on human intestinal Caco-2 cells and ex vivo on human serum. Both assays were applied to investigate the inhibitory activity of soy and lupin peptides. The best conditions for in situ DPP-IV activity in Caco-2 cells were obtained using 2-day cells and 50 µM Gly-Pro-AMC. Sitagliptin, used as reference inhibitor, showed a dose-dependent response with a 50% inhibition concentration (IC_50_) of 0.6 µM. A lower IC_50_ (0.2 µM) was obtained for sitagliptin on human serum incubated with the substrate for 24 h. Both assays were applied to assess the activity of Lup1 (LTFPGSAED) and Soy1 (IAVPTGVA) on DPP-IV. Lup1 and Soy1 inhibited DPP-IV in situ, with IC_50_ values of of 207.5 and 223.2 µM, respectively, and maintained their inhibitory activity ex vivo on circulating DPP-IV with a slightly lower potency. These assays can be used to characterize the DPP-IV inhibitory activity of food-derived molecules more accurately than in vitro biochemical tests. This combined approach also considers their effects on the circulating form of DPP-IV, correlated to metabolic diseases.

## 1. Introduction

Dipeptidyl peptidase IV (DPP-IV)/CD26 is a cell surface ectoenzyme (EC 3.4.14.5) that cleaves dipeptides from the N-terminus of polypeptides in which proline is at the penultimate position. DPP-IV, originally characterized as a T-cell differentiation antigen, was later reported to be ubiquitously expressed on the surface of various cell types, such as renal proximal tubules, intestinal epithelial cells, vascular endothelium, biliary caniliculi, alveolar pneumocytes, and skin fibroblasts [1]. A cleaved form of the enzyme, possessing similar activity, is also found in serum, released from the membrane by specific proteolytic cleavage to produce the soluble form. Enhanced soluble DPP-IV levels and/or activity have been suggested to be a novel regulator of many metabolic diseases, such as type 2 diabetes (T2DM), obesity, cardiovascular disease, and non-alcoholic fatty liver disease [2,3]. The importance of DPP-IV as a promising therapeutic target for glycemic control has recently emerged [4]. It plays a major role in glucose metabolism by N-terminal truncation and inactivation of the incretins glucagon-like peptide (GLP-1) and gastrointestinal insulinotropic peptide (GIP). Together, GIP and GLP-1 stimulate insulin biosynthesis at the pancreatic level and are responsible for up to 70% of insulin secretion following a meal [5]. However, they are rapidly inactivated by DPP-IV action [6]. Inhibition of DPP-IV has been shown to promote insulin secretion and suppress glucagon release by prolonging the half-life of GLP-1 and GIP [6,7]. Oral administration of synthetic DPP-IV inhibitors to type 2 diabetic patients effectively reduces glycated hemoglobin levels [7]. The synthetic DPP-IV inhibitors currently in use have very few side effects, but their long-term safety remains unknown [8]. Several food-derived peptides have been characterized for their anti-diabetic activity [9,10] and many of them have been identified and found to act as promising DPP-IV inhibitors, potentially contributing to glycemic control [11,12,13,14]. 

However, in most of these studies, DPP-IV inhibitory activity has been evaluated only in silico or with in vitro assays. Indeed, a careful literature analysis has highlighted that the main approach for identifying food-derived peptides with DPP-IV inhibitory activity is the use of biochemical tools involving purified porcine or human DPP-IV enzymes and a standard substrate. These tests have the advantages of commercial application and the ability to screen a large number of samples in a relatively short time, but also the disadvantage of providing an insufficient characterization of their activity before performing expensive in vivo experimental studies. The development of specific in situ cell-based assays and ex vivo tools would fill the gap between the biochemical and in vivo studies.

As DPP-IV is abundantly expressed on the luminal surface of enterocytes, any potential inhibitor deriving from food digestion is likely to first interact with intestinal DPP-IV and other intestinal peptidases before being absorbed, possibly being further metabolized, and released into circulation, where it can interact with soluble and vascular endothelial DPP-IV, thus affecting circulating GIP and GLP-1 levels. Thus, an intestinal cellular assay for DPP-IV inhibitory activities would be more advantageous, compared to an in vitro test, to assay the enzyme in its natural environment and to account for possible metabolic and transport effects. 

The human intestinal Caco-2 cell line is the best currently available and most widely used model of human intestinal enterocytes. From the time the cells reach confluence, they progressively differentiate within 14 to 21 days, expressing morphological and functional characteristics of mature absorptive enterocytes [15]. Several intestinal enzymes involved in food digestion are expressed on the surface of Caco-2 cells, including DPP-IV [16,17]. 

It is also important to underline that a soluble form of DPP-IV lacking the cytoplasmic and transmembrane domains is present in plasma and other body fluids [18] and that emerging evidences suggest a potential role for this circulating peptidase in the pathophysiology of metabolic and cardiovascular diseases [19]. In order to provide relevant information regarding the stability and ability of absorbed food-derived peptides, it would be certainly useful to develop a suitable ex vivo experiment on circulating DPP-IV using human serum samples.

Considering all of these observations, in order to evaluate the effect of food-derived peptides on circulating DPP-IV activity, ex vivo experiments were also developed in the present work using human serum samples. The aims of this study were: (1) to develop fast, sensitive, and accurate DPP-IV assays either in situ on human intestinal cells or ex vivo on human serum, using a fluorescent substrate and the specific DPP-IV inhibitor sitagliptin, and (2) to validate the assay on previously identified soy and lupin peptides with known DPP-IV inhibitory activity. In particular, two peptides were chosen: one deriving from lupin beta-conglutin precursor, Lup1 (LTFPGSAED), also named P7 [20,21], and the second from soy glycinin, Soy1 (IAVPTGVA) [12].

## 2. Materials and Methods

### 2.1. Chemicals

Dulbecco’s modified Eagle medium (DMEM) was obtained from GIBCO (Thermo Fisher Scientific, Waltham, MA USA). Fetal bovine serum (FBS) was from Hyclone Laboratories (Logan, UT, USA). Stable L-glutamine, 1% non-essential amino acids, penicillin/streptomycin, and PBS were from Euroclone (Milan, Italy). Sitagliptin and Gly-Pro-amido-4-methylcoumarin hydrobromide (Gly-Pro-AMC) were from Sigma-Aldrich (St. Louis, MO, USA). Polycarbonate filters, 12 mm in diameter, 0.4 m in pore diameter, were from Transwell Corning Inc. (Lowell, MA, USA). Peptides Lup1 (LTFPGSAED) and Soy1 (IAVPTGVA) were synthetized by the company PRIMM (Milan, Italy) at >95% purity. Human serum was freshly collected from a healthy female volunteer. 

### 2.2. Cell Culture

Caco-2 cells, obtained from INSERM (Paris, France), were routinely sub-cultured at 50% density [22] and maintained at 37 °C in a 90% air/10% CO_2_ atmosphere in DMEM containing 25 mM of glucose, 3.7 g/L of NaHCO_3_, 4 mM of stable L-glutamine, 1% nonessential amino acids, 100 U/L of penicillin, and 100 µg/L of streptomycin (complete medium), supplemented with 10% heat-inactivated fetal bovine serum (FBS; Hyclone Laboratories, Logan, UT, USA). 

### 2.3. In Situ Cell-Based DPP-IV Activity Assay Optimization

For the experiments, cells were seeded on black 96-well plates with clear bottoms at a density of 5 × 10^4^ cells/well. The medium was regularly changed three times a week. Caco-2 cells DPP-IV activity was assayed on days 2, 4, and 6 from seeding. On the day of the experiment, cells were washed once with 100 µL of PBS without Ca^++^ and Mg^++^. Then, 100 µL of Gly-Pro-AMC substrate at concentrations of 25, 50, 100, 200, and 400 µM in PBS without Ca^++^ and Mg^++^ were added and the fluorescence signals (excitation/emission wavelengths 350/450 nm) in each well were measured using a Synergy H1 (BioTek Instruments, Winooski, VT, USA) every 1 min, for up to 10 min.

### 2.4. In Situ Evaluation of the Inhibitory Effect of Sitagliptin, Lup1, and Soy1 on In Situ DPP-IV Activity

A total of 5×10^4^ Caco-2 cells/well were seeded in black 96-well plates with clear bottoms. The second day after seeding, spent media was discarded and the cells were treated with 100 µL of sitagliptin (from 10^−8^ to 10^−4^ M), Lup1 and Soy1 (from 10^−6^ to 10^−2.5^ M), or vehicle in growth medium for 1 h at 37 °C. Afterwards, treatments were removed and Caco-2 cells were washed once with 100 µL of PBS without Ca^++^ and Mg^++^, before the addition to each well of 100 µL of Gly-Pro-AMC substrate at the concentration of 50.0 µM in PBS without Ca^++^ and Mg^++^. Fluorescence signals (ex./em. 350/450 nm) were recorded using a Synergy H1 microplate reader every 1 min for up to 10 min.

### 2.5. Ex Vivo DPP-IV Activity Assay

Human serum samples prepared from freshly collected venous blood from a healthy female volunteer were aliquoted 40 µL/well in black 96-well plates and 10 µL of each inhibitor were added for 24 h at 37 °C. The inhibitors tested were sitagliptin (from 10^−9^ to 10^−3^ M), Lup1 and Soy1 (10, 100, and 300 µM). Subsequently, 50 µL of 100 µM Gly-Pro-AMC was added to each well to achieve a final substrate concentration of 50 µM. Fluorescence signals (ex./em. 350/450 nm) were then recorded using a Synergy H1 microplate reader every 1 min for up to 10 min.

### 2.6. Statistical Analysis

Statistical analyses were carried out by one-way ANOVA (Graphpad Prism 7, GraphPad Software, La Jolla, CA, USA) followed by Dunnett’s test. Values were expressed as means ± SD; *p*-values < 0.05 were considered to be significant.

## 3. Results

### 3.1. Development and Optimization of a Caco-2 Cell-Based DPP-IV Assay

In order to develop and optimize the experimental conditions for the in situ Caco-2 cell-based DPP-IV activity assay, two parameters were considered: cell time in culture and DPP-IV substrate concentration. DPP-IV is an enzyme also expressed by non-differentiated Caco-2 cells, although it increases during culture differentiation [16,17].

**Figure 1:**
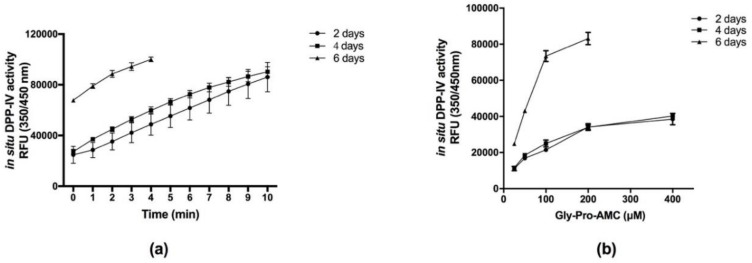
In situ DPP-IV activity on 2-, 4-, and 6-day Caco-2 cells: (**a**) using 50 µM Gly-Pro-AMC as a substrate, the fluorescence signal increased as a function of time and was linear at all cell ages over the first 3 min. (**b**) The fluorescence signal was measured after 3 min as a function of substrate Gly-Pro-AMC concentration at all cell ages. Data are the means ± SD of three experiments performed in triplicate.

Taking this into consideration, cells were seeded in 96-well plates at a high density (5 × 10^4^ cells/well) to rapidly achieve confluence and they were then cultured for 2, 4, and 6 days. Gly-Pro-AMC was selected as the specific fluorescent substrate for DPP-IV activity, since DPP-IV selectively metabolizes this substrate, releasing the AMC fluorophore, which emits fluorescence signals at 450 nm upon excitation at 350 nm. DPP-IV activity was tested after 2, 4, and 6 days old Caco-2 cells cultures, using 50 µM Gly-Pro-AMC in PBS, and the fluorescence signal was monitored up to 10 min. Figure 1a shows that the detected relative fluorescence units (RFUs) steadily increased as a function of time, reaching an overflow of the fluorescent signal at different times depending on the level of DPP-IV activity at each age of the cells. Subsequently, DPP-IV activity was tested as a function of substrate concentration (from 25 to 400 µM), using an appropriate time interval to be in the linear portion of the enzyme activity, avoiding signal overflow.

Figure 1b shows the detected RFUs after 3 min, for all cell ages at each tested substrate concentration. The results indicate that DPP-IV activities expressed by cells after 2 and 4 days of culture are similar, whereas a marked increase in activity was observed in cells after 6 days of culture. RFU values obtained after 6 days were in fact higher than those measured after 2 and 4 days at all tested substrate concentrations. Fluorescence steadily increased with Gly-Pro-AMC concentration, reaching a plateau at 200 µM in cells of all ages. 

Considering that cells aged 2 and 4 days are equivalent, and since the cells were certainly not differentiated, whereas at 6 days the differentiation started giving non-reproducible responses, it was decided to continue the experimentation using 2-day Caco-2 cell cultures and a concentration of 50 µM Gly-Pro-AMC as the optimal parameters. This saved time and money.

### 3.2. Sitagliptin Inhibits DPP-IV Activity in Caco-2 Cells

**Figure 2:**
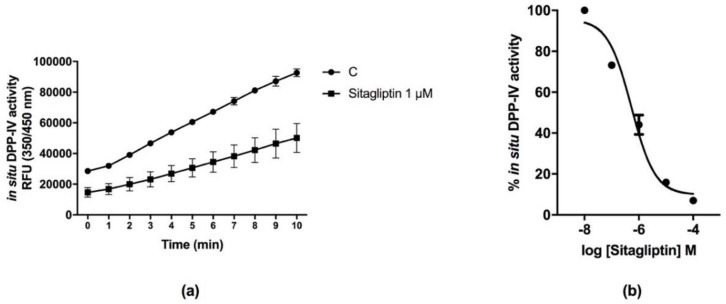
Inhibitory activity of sitagliptin on DPP-IV activity measured in situ on 2-day Caco-2 cells: (**a**) at 1 µM, sitagliptin inhibited DPP-IV activity in Caco-2 cells by over 50%; (**b**) sitagliptin inhibited DPP-IV activity in a dose-response manner, exhibiting an IC_50_ of 0.6 µM. Data are the means ± SD of three experiments performed in triplicate.

Sitagliptin is a known synthetic DPP-IV inhibitor that was used as a reference compound for the validation of the Caco-2 cell-based DPP-IV activity assay. Figure 2 shows the inhibitory effect of DPP-IV activity by sitagliptin in situ. In detail, Caco-2 cells after 2 days of culture were treated for 1 h with sitagliptin at the fixed concentration of 1 µM, the Gly-Pro-AMC (50 µM) was added and the fluorescence signals were measured for 10 min. The increase of the fluorescence signals, corresponding to DPP-IV activity, was reduced by over 50% in the presence 1.0 µM sitagliptin compared to the control (Figure 2a). To obtain information on the efficiency of DPP-IV inhibition, sitagliptin concentrations ranging from 10^−8^ to 10^−4^ M were tested. The results indicated that sitagliptin inhibited DPP-IV activity in 2-day Caco-2 cells in a dose-dependent manner, displaying a 50% inhibitory concentration (IC_50_) value of 0.6 µM (Figure 2b). Similar experiments were performed in 4- and 6-day Caco-2 cells: the IC_50_ values at 4 and 6 days were 0.4 and 0.65 µM, respectively (Figure S1).

### 3.3. Peptides Lup1 and Soy1 Inhibit DPP-IV Activity Expressed in Caco-2 Cells

**Figure 3:**
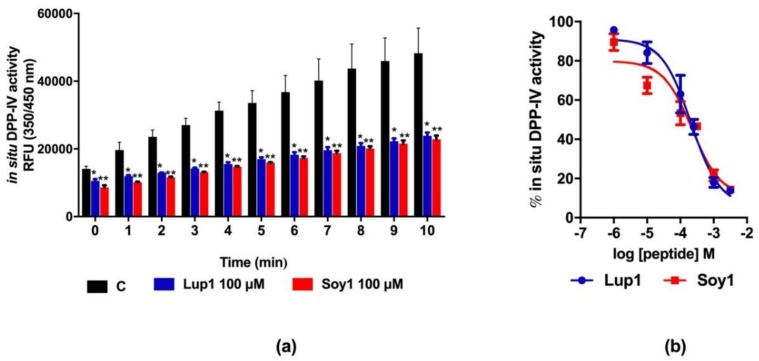
Inhibition of in situ DPP-IV activity by Lup1 and Soy1: (**a**) after pre-incubation (1 h) with 100 µM Lup1 and Soy1, DPP-IV activity was inhibited by 50% by both peptides; (**b**) measuring inhibitory activity as a function of peptide concentration, a dose-dependent response was observed with IC_50_ values of 207.5 µM for Lup1 and 223.2 µM for Soy1. Data are the means ± SD of three experiments performed in triplicate. *: *p* > 0.05; **: *p* > 0.01.

Lup1 and Soy1 have previously been shown to exhibit DPP-IV inhibitory activity by an in vitro assay, using a purified human recombinant enzyme with IC_50_ values of 228.0 and 106.0 µM, respectively [12]. The validated Caco-2 cell-based DPP-IV activity assay was used to test the effects of both peptides on DPP-IV activity in situ. Initially, Caco-2 cells were treated with Lup1 and Soy1 at the fixed concentration of 100 µM for 1 h, then Gly-Pro-AMC substrate was added and the reaction mixture was incubated for 10 min, during which the fluorescent signal was monitored every 1 min. The results indicated that during the incubation time, a linear increase of RFU values was observed, corresponding to the in situ DPP-IV activity. This in situ DPP-IV activity was reduced by 50% after 1 h of pre-incubation with both peptides as compared to the control samples (Figure 3a). Afterwards, in order to calculate the IC_50_ values, Lup1 and Soy1 concentrations ranging from 10^−6^ to 10^−2.5^ M were tested. Both peptides inhibited the DPP-IV activity in Caco-2 cells in a dose-dependent manner, displaying IC_50_ values of 207.5 and 223.2 µM, respectively (Figure 3b).

### 3.4. Circulating DPP-IV Inhibition by Peptides Lup1 and Soy1

**Figure 4:**
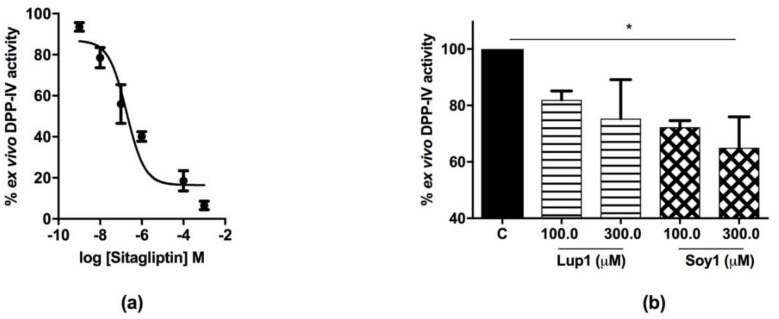
Ex vivo assay of circulating DPP-IV in human serum: (**a**) sitagliptin showed a dose-dependent inhibition of circulating DPP-IV with an IC_50_ of 0.2 µM; (**b**) Lup1 inhibited circulating DPP-IV activity by 18.1% and 24.7% at 100 µM and 300 µM, respectively. Soy1, at the same concentrations, showed a slightly higher inhibitory activity of 27.7% and 35.0%, respectively. Data are the means ± SD of three experiments performed in triplicate. *: *p* > 0.05.

In order to develop a method to evaluate the effect of peptides Lup1 and Soy1 on circulating DPP-IV activity, ex vivo experiments were performed using human serum samples and sitagliptin as a reference inhibitor. In detail, human serum was incubated for 24 h with different concentrations of sitagliptin ranging from 10^−9^ to 10^−3^ M. At the end of the incubation time, the substrate was added and the fluorescence measured. Figure 4a shows that sitagliptin is able to inhibit circulating DPP-IV activity in a dose-dependent manner with an IC_50_ of 0.2 µM. Similarly, the peptides Lup1 and Soy1 were incubated with serum samples at 100.0 and 300.0 µM, respectively, for 24 h at 37 °C, to assess their activity on circulating serum DPP-IV. The findings clearly suggested that both peptides maintain their ability to inhibit the DPP-IV activity ex vivo (Figure 4b). Specifically, peptide Lup1 decreased the DPP-IV activity in the serum by 18.1% and 24.7%, whereas Soy1 reduced the circulating enzyme activity by 27.7% and 35.0% at 100.0 and 300.0 µM concentrations, respectively, versus the control samples (Figure 4b). 

## 4. Discussion

### 4.1. Development and Validation of a Cell-Based DPP-IV Activity Assay Using Human Intestinal Caco-2 Cells and an Ex Vivo Assay on Circulating DPP-IV Activity in Human Serum

In order to screen and identify novel food-derived DPP-IV inhibitors, the exclusive use of biochemical tools represents a major limitation for the lack of several factors that might influence their activity [23]. Moreover, since in vivo evidence of their potential activity as DPP-IV inhibitors is scarce, the development of an alternative and cost-effective strategy is needed. For this reason, the optimization of a cell-based DPP-IV activity assay represents an important target to fill this relevant gap. Human Caco-2 cells represent an appropriate intestinal cell culture model expressing several morphological and functional features of small intestinal enterocytes [15]. Furthermore, Caco-2 cells express good levels of DPP-IV mRNAs, which translate to active DPP-IV protein levels on the Caco-2 apical cell membrane [16,17], facing the intestinal lumen. From a physiological point of view, the intestinal epithelial cell layer is the first major barrier to absorption encountered by food-derived bioactive peptides. In particular, the protease activities, located both on the enterocyte surface and intracellularly, may affect the stability and integrity of food-derived peptides, which could be degraded and/or modified, affecting either their transport across the intestinal epithelium or their biological activity. Based on all of these considerations, the evaluation of the DPP-IV inhibitory activity of food-derived peptides on the intestinal cells has additional advantages, compared to the traditionally-used in vitro assay on the purified enzyme, as it mimics the intestinal environment as well as its transport and metabolic activities. Apically-expressed enzyme activities can reliably and efficiently be measured in live Caco-2 cells differentiated on filter inserts [24]. 

A similar cell-based assay has been recently proposed by other authors to evaluate DPP-IV activity in living 7-day-differentiated Caco-2 cells [23]. Our method is more sensitive and cost-effective, since here undifferentiated 2-day Caco-2 cells are used and a much lower concentration of the substrate Gly-Pro-AMC is employed with respect to the previous method (50 µM versus 1 mM) [23]. Since the fluorescent substrate concentration and level of enzyme expressed as a function of cell culture age are tightly connected, the optimized conditions here proposed are a good compromise to save time and money. In addition, a reference inhibitor of DPP-IV, sitagliptin, was used to validate the specificity of the in situ assay. Interestingly, the IC_50_ values of sitagliptin were found to be similar in Caco-2 cells after 2, 4, and 6 days, indicating that the characteristics of the enzyme in Caco-2 cells do not change as a function of time in culture, thus justifying the use of undifferentiated 2-day Caco-2 cells.

As mentioned in the introduction, besides the DPP-IV expressed on the cellular membranes, the soluble form of this enzyme is also positively associated with the development of metabolic diseases. For this reason, the soluble form of DPP-IV is also an important target for the management of type 2 diabetes (T2DM). In this context, it is not so obvious that, after digestion and absorption, food-derived peptides might retain their ability to also inhibit the circulating DPP-IV target. For this reason, another assay was developed and validated to assess ex vivo the inhibitory activity on the circulating form of DPP-IV using human serum. Again, the reference inhibitor sitagliptin was used to validate this ex vivo assay, because it is known that this drug is rapidly absorbed in vivo and acts on circulating and endothelial-expressed DPP-IV [25]. The ex vivo IC_50_ of sitagliptin was of the same order of magnitude of the value obtained in the in situ assay, both in the lower µmolar range. The fact that this ex vivo approach can also be applied to investigate the activity of a DPP-IV inhibitor has an important physiological relevance, since circulating DPP-IV is mainly responsible for the degradation of glucagon-like peptide-1(GLP-1) and GIP incretins, regulating plasma glucose levels [3,26].

As indicated in the introduction, the exclusive usage of the biochemical screening represents a main limitation of this very dynamic field. This relevant aspect should be carefully considering while selecting the best food-derived peptides for further in vivo and clinical investigations. The methodologies provided here may be useful to fill the gap between the biochemical assays and in vivo studies.

### 4.2. Lup1 and Soy1 Inhibit DPP-IV Activity

DPP-IV inhibitors have emerged as a new class of oral antidiabetic agents [27], with excellent therapeutic potentials in the management of T2DM [28,29,30]. Although synthetic drugs such as sitagliptin and vildagliptin are successfully commercialized, in the last years relevant studies have been focusing on food-derived peptides as novel natural DPP-IV inhibitors [31,32].

In this context, many of these food peptides and/or food-protein hydrolysates are mainly screened and characterized using in vitro and in silico approaches. Recently, we identified two lupin and soybean peptides with a DPP-IV inhibitory activity: in particular, using an in vitro DPP-IV activity assay, peptides Lup1 and Soy1 were shown to be able to inhibit the enzyme activity with a dose response behavior, displaying IC_50_ values of 228.0 and 106.0 µM, respectively [25]. Moreover, a molecular docking analysis permitted the prediction of the key molecular interactions, which stabilize the active conformations of Lup1 and Soy1 within the DPP-IV enzyme site.

However, in the field of food-derived peptides with DPP-IV inhibitory activity, an important limitation derives from the prevalent use of biochemical techniques, in which only the purified DPP-IV enzyme is used to screen and characterize their bioactivity. In fact, the in vitro enzymatic assays do not account for several parameters that might influence peptide bioactivity, such as resistance to digestive enzymes or peptide absorption. Thus, there is a need for an improved characterization of the inhibitory property of food-derived peptides using assay models that take into account intestinal environmental parameters that could modify peptide bioactivity, such as the proteolytic activity of the brush border (BB). 

Considered all of these aspects, using live intestinal Caco-2 cells, experiments were performed to investigate in detail the ability of Lup1 and Soy1 to inhibit DPP-IV activity, in the presence of BB proteolytic activity. Our findings clearly suggested that both peptides are able to reduce the enzyme activity, maintaining the dose-response relation. Specifically, the calculated IC_50_ of Lup1 was in agreement with that obtained with the in vitro assay, whereas that of Soy1 was 2-fold higher than that obtained by the in vitro tool. These results underline the different behavior and stability of these peptides in the presence of the complex intestinal environment, as we have previously reported [20,33], and reflect the different DPP-IV inhibiting activities under intestinal-like conditions. In particular, we have previously demonstrated that Lup1 peptide is absorbed intact by differentiated intestinal Caco-2 cells and it is not degraded in the presence of apical proteases after 4 h of incubation [20], a fact that can explain why it exhibited an IC_50_ that was in line with that obtained in vitro [12]. On the contrary, Soy1 is susceptible to the action of intestinal endopeptidases in Caco-2 cells, which results in the production of breakdown fragments, such as IAVPT and AVPTGVA, already after 2 h of incubation [33]. Thus, the reduced DPP-IV inhibitory activity of Soy1 detected by the cell-based technique is likely to be due to its metabolic degradation in situ by the hydrolytic activity of BB membrane peptidases. As already indicated above, the DPP-IV inhibitory properties of absorbed food-derived peptides may act on the circulating form of this enzyme. The inhibitory activity of Lup1 and Soy1 was therefore tested ex vivo on human serum, showing that both peptides retained their activity on the circulating serum DPP-IV, although at a slightly lower potency than observed in vitro [12] and in situ.

## 5. Conclusions

In conclusion, to the best of our knowledge, these are the first food-derived peptides extensively investigated for their behavior on the cell membrane and circulating forms of DPP-IV. This experimental approach, combining in situ and ex vivo DPP-IV assays, could be applied to study the inhibitory potential of other food-derived peptides in a more realistic fashion, thus overcoming the use of more expensive and less ethical in vivo approaches on experimental animals.

## Supplementary Materials

The following are available online at http://www.mdpi.com/2072-6643/10/8/1082/s1, Figure S1: In 4- and 6-day Caco-2 cells, sitagliptin inhibits DPIV activity in a dose-response manner with IC_50_ values of 0.4 and 0.65 µM, respectively. Data are the means ± SD of three experiments performed in triplicate.

## Abbreviations

AMC	amido-4-methylcoumarin hydrobromide
BB	brush border
DMEM	Dulbecco’s modified Eagle’s medium
DPP-IV	dipeptidyl peptidase IV
FBS	fetal bovine serum
GLP-1	glucagon-like peptide-1
GIP	gastrointestinal insulinotropic peptide
IC_50_	50% inhibitory concentration
PBS	phosphate-buffered saline
RFU	relative fluorescence unit
T2DM	type 2 diabetes
