# Supplementary figures and images for: Soybean- and Lupin-Derived Peptides Inhibit DPP-IV Activity on In Situ Human Intestinal Caco-2 Cells and Ex Vivo Human Serum
Source: Nutrients. 2018 Aug 13;10(8):1082. doi: 10.3390/nu10081082 (PMC6115767; doi:10.3390/nu10081082)

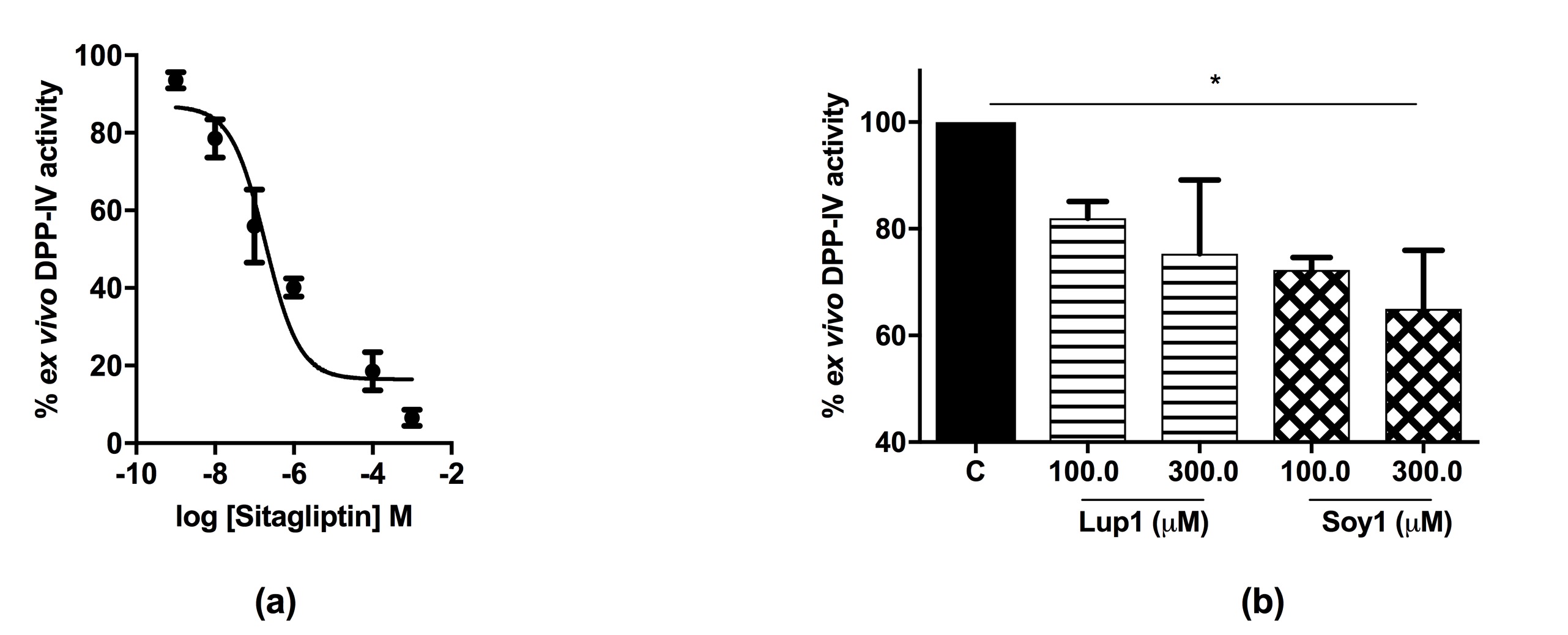

Supplement: Supplementary file 1 [file nutrients-10-01082-s001.zip › Figure 4 nut.jpg]

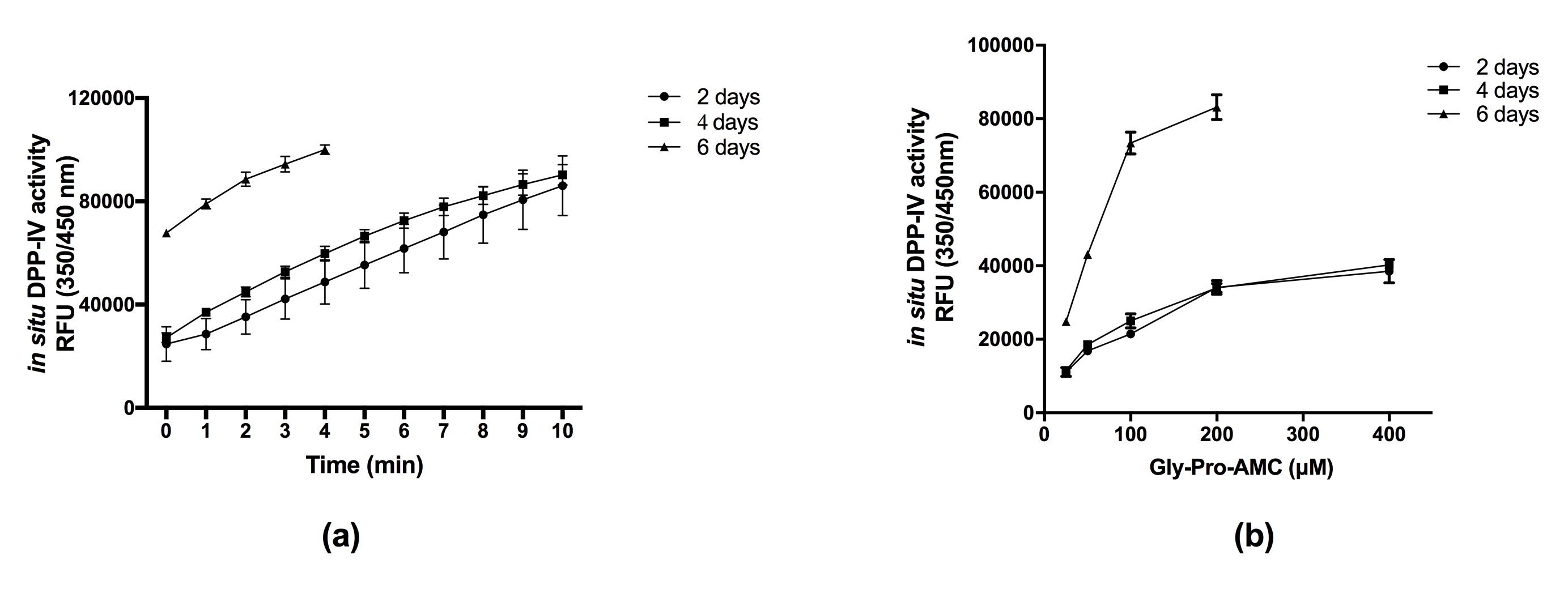

Supplement: Supplementary file 1 [file nutrients-10-01082-s001.zip › Figure 1 nut.jpg]

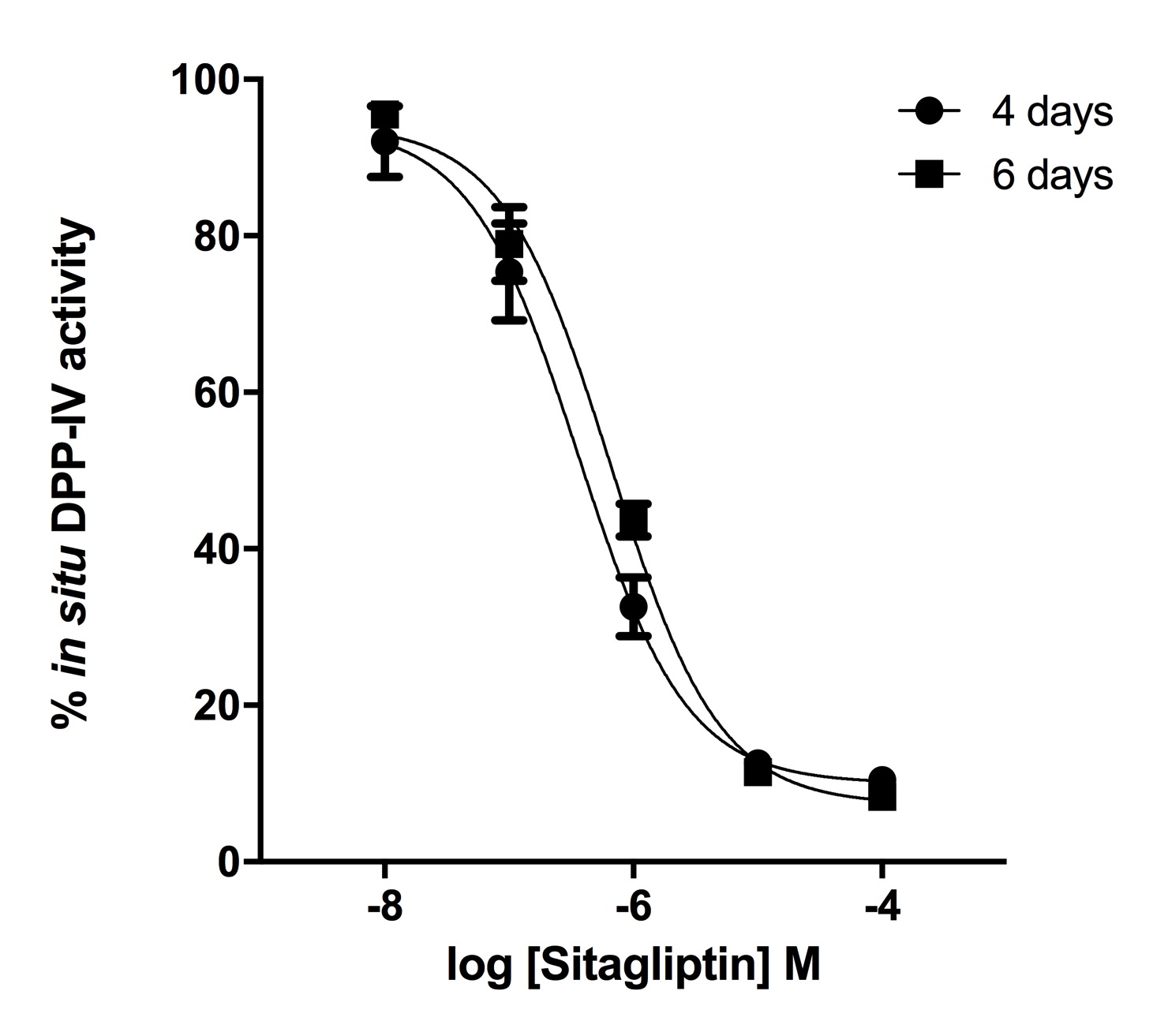

Supplement: Supplementary file 1 [file nutrients-10-01082-s001.zip › Figure 1S.jpg]

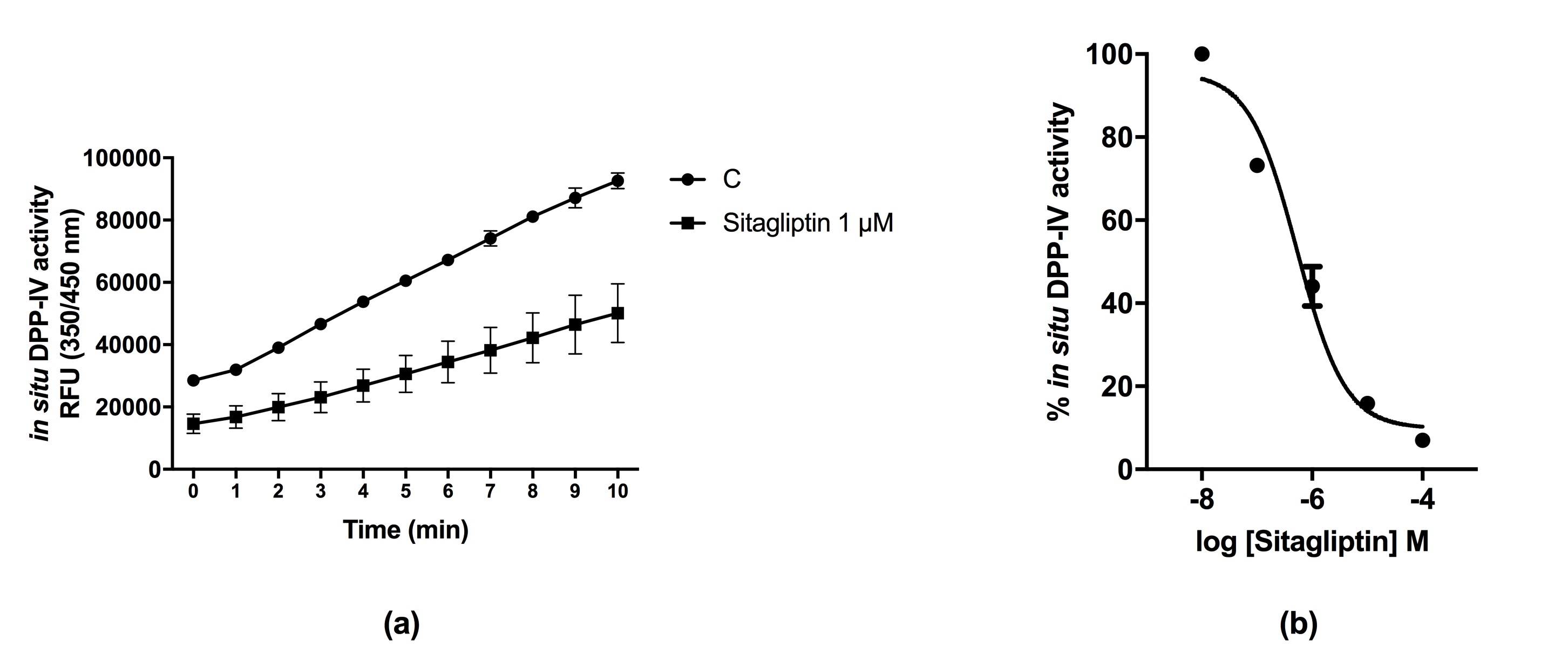

Supplement: Supplementary file 1 [file nutrients-10-01082-s001.zip › Figure 2 nut.jpg]

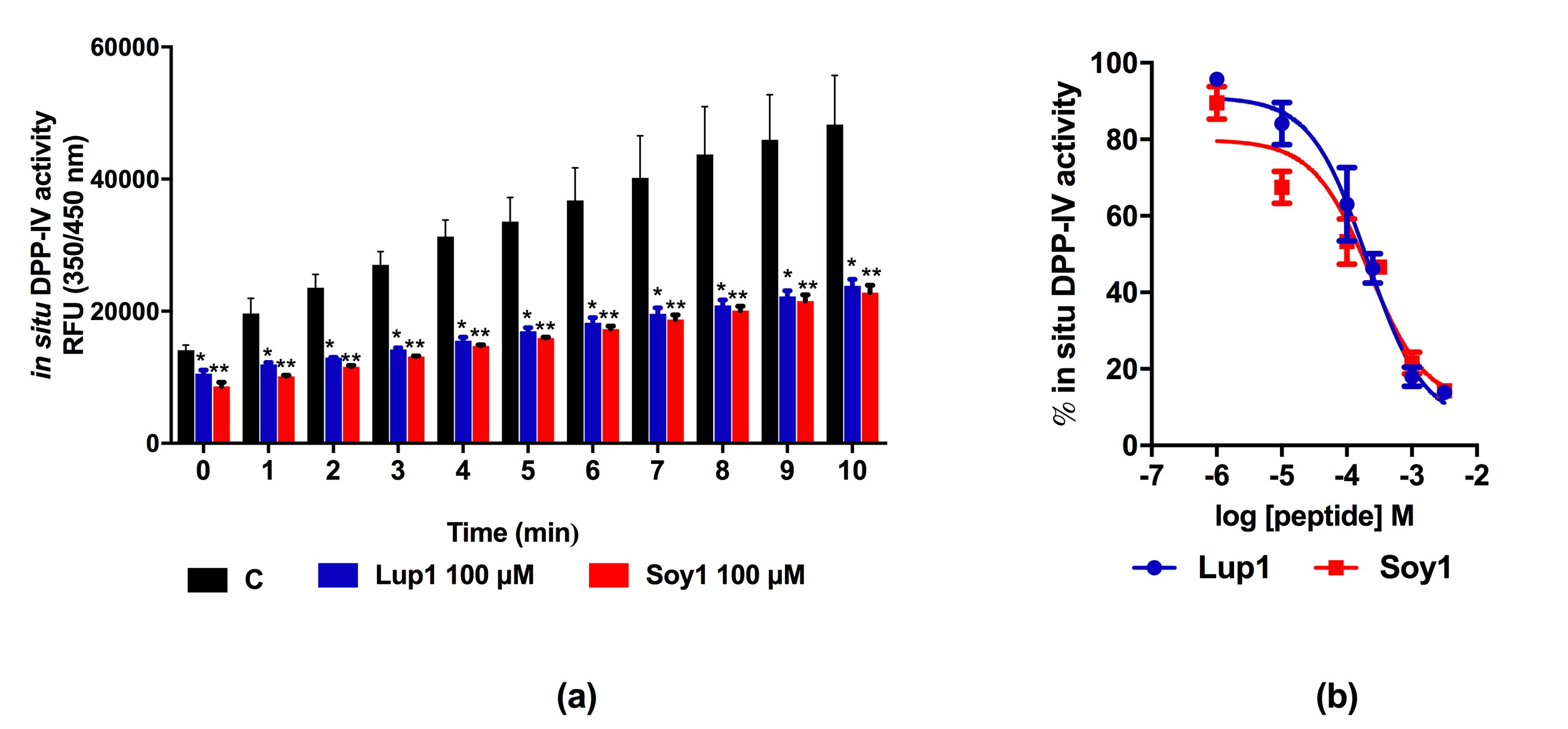

Supplement: Supplementary file 1 [file nutrients-10-01082-s001.zip › Figure 3 nut.jpg]
